# P01-013 – Cochlear involvement in FMF

**DOI:** 10.1186/1546-0096-11-S1-A17

**Published:** 2013-11-08

**Authors:** N Aktay Ayaz, G Keskindemirci, A Batıoglu, G Aydogan, E Aldemir, Z Donmez, O Yigit, A Akçay, S Ozen

**Affiliations:** 1 Pediatric Rheumatology, Istanbul Kanuni Sultan Suleyman Education and Research Hospital, Turkey; 2 Otolaryngology Head and Neck Surgery, Istanbul Education and Research Hospital, Turkey; 3 Pediatric ematology, Istanbul Kanuni Sultan Suleyman Education and Research Hospital, Istanbul; 4 Pediatric Rheumatology, Hacettepe University Medical Faculty, Ankara, Turkey

## Introduction

FMF is a monogenic autoinflammatory disease with recurring episodes of fever and serositis attacks. FMF is associated with mutations in pyrin. On the other hand mutations in a molecule in the same pathway, cryopyrin, is characterized by inflammatory features involving the inner ear as well. A study has suggested the involvement of cochlea in Behçet disease, which is a polygenic autoinflamatory disease.

## Objectives

To evaluate the cochlear function of children with the diagnosis of FMF prospectively.

## Methods

Children included to the study were diagnosed as FMF according to previously suggested criteria. Forty-three children with FMF and 20 controls were enrolled to the study. Demographic data and MEFV mutation analysis were recorded. Patients with any middle and external ear pathology were excluded from the study. After otoscopic inspection, audiometric examinations were carried out including otoacustic emission testing by distortion products (DP) and signal noise ratio (SNR) testing with 1000, 1400, 2000, 2800 and 4000 Hz and audiometric evalution including pure tone average (PTA) measurements with high frequency levels that were 8000, 10000, 12500, 16000 Hz. The results of cochlear function evaluations of the patients and controls were analysed.

## Results

The patient group included 43 children (27 female and 16 male patient) with mean age 11.9 (range 26 months-18 years) and the control group was age and sex matched. PTA levels were normal in both FMF patients and the control group. However, hearing levels at the frequency of 10000 Hz was found to be significantly higher in the FMF group (p<0.05). In otoacoustic emission evalution, SNR of the FMF group was lower in frequency at 1000 Hz (p<0,05).

## Conclusion

Even though hearing function was normal there were a number of abnormalities especially at higher frequencies like 10000 Hz. Our results need to be confirmed in larger groups. Further studies are needed to understand whether these subtle changes are significant and whether they are due to subclinical inflammation of FMF.

## Disclosure of interest

None declared

